# Probiotics and carriage of *Streptococcus pneumoniae* serotypes in Danish children, a double-blind randomized controlled trial

**DOI:** 10.1038/s41598-018-33583-9

**Published:** 2018-10-15

**Authors:** Sine Fjeldhøj, Rikke Pilmann Laursen, Anni Larnkjær, Christian Mølgaard, Kurt Fuursted, Karen Angeliki Krogfelt, Hans-Christian Slotved

**Affiliations:** 1 Department of Bacteria, Parasites and Fungi, Statens Serum Institut, Copenhagen, 2300 Denmark; 2 Department of Nutrition, Exercise and Sports, Faculty of Science, University of Copenhagen, Copenhagen, 2200 Denmark

## Abstract

This study examined the carriage of *Streptococcus pneumoniae* in healthy Danish children aged 8–19 months and assessed the effect of the probiotics *Lactobacillus rhamnosus* GG and *Bifidobacterium animalis* subsp *lactis* on the pneumococcal carriage during daycare enrolment. Potential risk factors of pneumococcal carriage were analysed and the carriage study was compared with registered invasive pneumococcal disease (IPD) data. This study is a part of the ProbiComp study, which was a double-blind, randomized controlled trial, including 290 children allocated to probiotics or placebo for 6 months and recruited during two autumn seasons (2014/2015). Pneumococci were identified by optochin sensitivity, bile solubility, α-hemolysis and/or capsular reaction. Serotyping was performed by latex agglutination kit and Quellung reaction. The carriage rate of *S. pneumoniae* was 26.0% at baseline and 67.4% at the end of intervention. No significant difference was observed between the placebo group and the probiotics group (p = 0.508). Children aged 8–19 months were carriers of non-pneumococcal vaccine serotypes causing IPD in children aged 0–4 years. However, serotypes causing most IPD cases in Danish elderly were either not found or found with low prevalence suggesting that children are not the main reservoir of those serotypes and other age groups need to be considered as carriers.

## Introduction

*Streptococcus pneumoniae* can cause invasive pneumococcal disease (IPD) which worldwide is associated with high mortality and morbidity at all ages despite the use of effective vaccines^1^. *S. pneumoniae* colonizes the epithelium of the nasopharynx and at least 92 different serotypes are known^2,3^. *S. pneumoniae* can cause meningitis, otitis media, pneumonia, sinusitis and bacteremia primarily in young children and elderly^4^.

Carriage of *S. pneumoniae* is a prerequisite for developing IPD, and is most frequent in young children, who act as reservoirs^4^. High carriage rate is associated with a high prevalence of respiratory infections^5^ and it is believed that children transmit IPD serotypes to other age groups^6–8^. However, transmission from adults to children is also observed^8^. Potential risk factors of pneumococcal carriage include attending daycare, young age, having siblings in daycare, having siblings <5 years and genetic and environmental factors such as socioeconomic conditions and passive smoking^4,9,10^. Daycare attendance is considered a major risk factor^9,11,12^. The currently available vaccines protect against a limited number of the known serotypes^3^. The 7-valent pneumococcal conjugate vaccine (PCV7) was included in the Danish Childhood Immunization Program in 2007 and was replaced by PCV13 in 2010^1^. PCV7 includes serotype 4, 6B, 9V, 14, 18C, 19F, and 23F while PCV13 includes the PCV7-serotypes and additional serotypes 1, 3, 5, 6A, 7F, and 19A^1,7^. PCV7 led to a significant reduction in IPD caused by PCV7-serotypes markedly among children aged <2 years^1,3,6,13^, but also a significant reduction in IPD cases and carriage of vaccine serotypes among older children and adults was seen, especially in the age group 65+ years^3,8^. This phenomenon is known as herd protection^8^. With the reduction of PCV-serotypes, an increase in the incidences of IPD caused by non-vaccine serotypes has been observed^1,6,7^. Thus, it is important to continue surveillance of serotype distribution.

Only a few pneumococcal carriage studies in children have been conducted in Denmark, the most recent was prior to the introduction of PCV7^9^.

The purpose of this study was to assess the effect of probiotics on *S. pneumoniae* carriage in healthy Danish children aged 8–19 months, determine serotype distribution and analyse risk factors for pneumococcal carriage. Furthermore, to assess whether the carried serotypes are also isolated in IPD cases of all age groups as described by Slotved *et al*. (2016)^14,15^ and Harboe *et al*.^16^.

## Methods

### Study population

This study is part of the ProbiComp study, which was a randomised, double-blind, placebo-controlled parallel study investigating the effect of probiotics on infections in young children starting daycare described in details in Laursen *et al*.^17^.

The ProbiComp study included 290 healthy children aged 8–13 months starting daycare within 12 weeks after start of intervention. They were randomly assigned to a combination of the probiotics *Lactobacillus rhamnosus* GG (LGG) and *Bifidobacterium animalis* subsp *lactis* (BB-12), administered orally, in a dose of 10^9^ colony-forming units/day (CFU/day) of each or placebo (maltodextrin) for 6 months. Children were examined at baseline before start of intervention and 6 months later at the end of intervention. The children were recruited during two autumn seasons from mid-August to mid-December in 2014 and 2015. LGG and BB-12 are registered trademarks of Chr. Hansen A/S. For exclusion criteria see Laursen *et al*.^17^. Study design, compliance, randomisation, data collection, outcome measurements and sample size calculation are described in Laursen *et al*.^17^. Briefly, both parents and study personnel were blinded to group allocation, and the placebo powder and the probiotics powder did not differ in smell, taste or colour. Parents registered daily whether the child had ingested the product and by the end of intervention the parents returned the registration sheets and unused sachets with powder to evaluate compliance. PCV13 is administered in a 2 + 1 dose schedule at the age of 3, 5 and 12 months^1^. At baseline 98.2% of the children examined were covered by at least one dose of PCV13, while 92.6% were covered by two doses. At the end of intervention 88.0% of the children were covered by all three doses of PCV13. Vaccination data were obtained at the Danish Vaccination Register (DDV) (record number 2015-57-0102). Information regarding the children’s background and health were obtained by interviewing parents and during the intervention period occurrence of children’s symptoms of illness, absence from daycare, doctor’s visits, and doctor-diagnosed illnesses were registered by the parents in weekly and daily web-based questionnaires^17^.

### Nasal swab sampling

Two autumn seasons were included in the study. At baseline, samples were analysed from 141 children from the first season (2014) and 144 children from the second season (2015). At the end of intervention, samples from 124 children from the first season and 134 children from the second season were analysed.

Nasal swab samples were collected by a modified version of Satzke *et al*.^18^. Minitip flocked swabs (FLOQSwabs™, Copan, Italy) were used to take the samples. The swabs were inserted as far as possible into the nasal cavity and rotated, although within the limitations of the children’s comfort. They were then placed in 1 mL Luria-Bertani (LB) broth with 10% glycerol in cryotubes, and stored at −80 °C until analysis.

### Identification of pneumococcal serotypes

Identification of pneumococcal serotypes was performed as previously described^4^. Briefly, 10 µL of each sample was added to 3 mL serum-ox broth and incubated overnight at 37 °C in 5% CO_2_, before plating. The following day 1 µL of each serum-ox broth was cultured on 10% horse blood agar plates, which were incubated overnight at 37 °C, 5% CO_2_. *S. pneumoniae* were identified based on optochin sensitivity, bile solubility, α-hemolysis and/or capsular reaction (Quellung reaction). Pneumotest latex agglutination kit (SSI Diagnostica, Hillerød, Denmark) was performed on the serum-broth to determine pneumococcal group. Serotypes were identified by the Quellung reaction (Neufeld test) using serotype specific antisera (SSI Diagnostica, Hillerød, Denmark). The specimens were screened by pneumotest latex agglutination kit for multiple serotypes. If multiple serotypes were found, they were isolated and serotyped.

### Invasive pneumococcal disease data

Data on invasive pneumococcal isolates in the period 2014–2016 were obtained from the Danish laboratory surveillance system at the National Neisseria and Streptococcus Reference Laboratory (NSR), Statens Serum Institut (SSI) as described by Slotved *et al*. (2016)^14,15^. Briefly, information on age and serotype was used. An IPD case was defined as *S. pneumoniae* occurring in normally sterile sites such as cerebrospinal fluid or blood. The coverage and evaluation of the database in Denmark was described by Harboe *et al*.^16^.

### Data analysis

RStudio version 1.0.136 and R version 3.4.1 for Windows was used for calculation of odds ratios (OR), confidence intervals (95% CI), and p-values using two tailed Fisher’s Exact Test (http://www.r-project.org/ last accessed: 02.20.2018). A p-value < 0.05 was considered significant. RStudio was also used to make graphical illustrations.

We examined several risk factors for pneumococcal carriage including sex, having siblings <5 years, breastfeeding at baseline examination, living with a dog or cat, passive smoking exposure in the household, having respiratory infections such as bronchitis, pneumonia or otitis media during the intervening period and the effect of receiving antibiotics during the intervening period of 6 months. We did not have access to dates on when antibiotics were received. Univariate logistic regression was not undertaken as confounders were evenly distributed during the randomization process, therefore multivariate logistic regression was not required. Crude odds ratio of pneumococcal carriage was estimated in each characteristic (sex, having siblings <5, breastfeeding, respiratory infections including bronchitis, pneumonia and otitis media, antibiotic use, passive smoking exposure and exposure to cats or dogs) using two-tailed Fisher’s exact test.

### Ethical considerations

The ProbiComp study protocol was approved by the Committees on Biomedical Research Ethics for the Capital Region of Denmark (H-4-2014-032), and we hereby confirm that all methods were performed according to the guidelines and regulations approved by the Committees on Biomedical Research Ethics. The study was registered at clinicaltrials.gov (identifier NCT02180581 (Supplementary file), posted 02/07/2014)^17^. Informed consent from parents and legal guardians of the children was required. Participation in the study was voluntary and parents could withdraw their consent at any time^17^.

Regarding IPD data, no ethical approval or informed consent was required since data were collected routinely for national surveillance purposes. Using the data is approved by the Danish Data Protection Agency (record number 2007-41-0229).

## Results

### Carriage study

The carriage rate of *S. pneumoniae* at baseline of the two seasons were comparable with no statistical significant difference between the two seasons as seen in Table 1. Hence, the two seasons were combined as one baseline group for further analysis. The mean carriage rate at baseline therefore was 26.0% (CI 20.8–31.1%) (Table 1).

**Table 1:** Comparison of carriage rate of the two seasons and a comparison of the placebo groups and the probiotics groups. Odds ratios (OR) and p-values were calculated by two-tailed Fisher’s exact test.

	Carriage rate, % (95% CI)	OR (95% CI)*	p-value*
Baseline			
Season 1 (2014)	27.7 (20.3–35.0%)	1.00	1.00
Season 2 (2015)	24.3 (17.3–31.3%)	0.840 (0.477–1.477)	0.589
End of intervention			
Season 1 placebo group	60.7 (48.4–72.9%)	1.00	1.00
Season 2 placebo group	69.6 (58.7–80.4%)	1.478 (0.674–3.268)	0.356
Season 1 probiotics group	76.2 (65.7–86.7%)	1.00	1.00
Season 2 probiotics group	63.1 (51.3–74.8%)	0.536 (0.229–1.228)	0.127
Placebo group	65.4 (57.2–73.6%)	1.00	1.00
Probiotics group	69.5 (61.6–77.5%)	1.207 (0.694–2.107)	0.508
Overall carriage rate			
Baseline^a^	26.0 (20.8–31.1%)	1.00	1.00
End of intervention^b^	67.4 (61.7–73.2%)	5.884 (4.007–8.715)	<0.001^c^

^a^Mean of season 1 (2014) and season 2 (2015). ^b^Mean of the placebo group and the probiotics group. ^c^Comparison of the baseline and the end of intervention. *Calculated by Fisher’s Exact Test in RStudio. CI: confidence intervals, OR: odds ratio.

At the end of intervention half of the children had received the probiotics LGG and BB-12 for 6 months, hence each season included both a placebo group and a probiotics given group (Table 1). The two seasons were alike with no statistical significant difference between the two placebo groups and the two probiotics groups, hence data were combined into one placebo group and one probiotics group (Table 1).

The carriage rate of *S. pneumoniae* in children receiving the probiotics LGG and BB-12 was 69.5% (CI 61.6–77.5%) while the carriage rate in the placebo group was 65.4% (CI 57.2–73.6%). Thus, probiotics do not have a significant effect on the pneumococcal carriage rate (p = 0.508) (Table 1). Furthermore, the serotype diversity/distribution in the probiotics group and placebo group were not significantly different (data not shown). The placebo group and the probiotics group were therefore combined into one group representing the end of intervention for further analysis. The carriage rate at the end of intervention was 67.4% (CI 61.7–73.2%), which is significantly higher than the carriage rate at baseline (p < 0.001) (Table 1).

All the cases of multiple serotype carriage involved two serotypes except one child at the end of intervention, who carried three serotypes (Table 2).

**Table 2:** Carriage rate and proportion of carriers with multiple serotypes.

	Baseline	End of intervention
Number of children analyzed	285	258
Age	8–13 months	14–19 months
Year of swab sample collection	2014 and 2015 (autumn)	2015 and 2016 (spring)
Carriage rate, % (N)	26.0 (74)	67.4 (174)
Proportion carriers with >1 serotype, % (N)	2.5 (7)	4.3 (11)
Proportion carriers with >2 serotype, % (N)	0 (0)	0.4 (1)

N: number of carriers.

**Figure 1:**
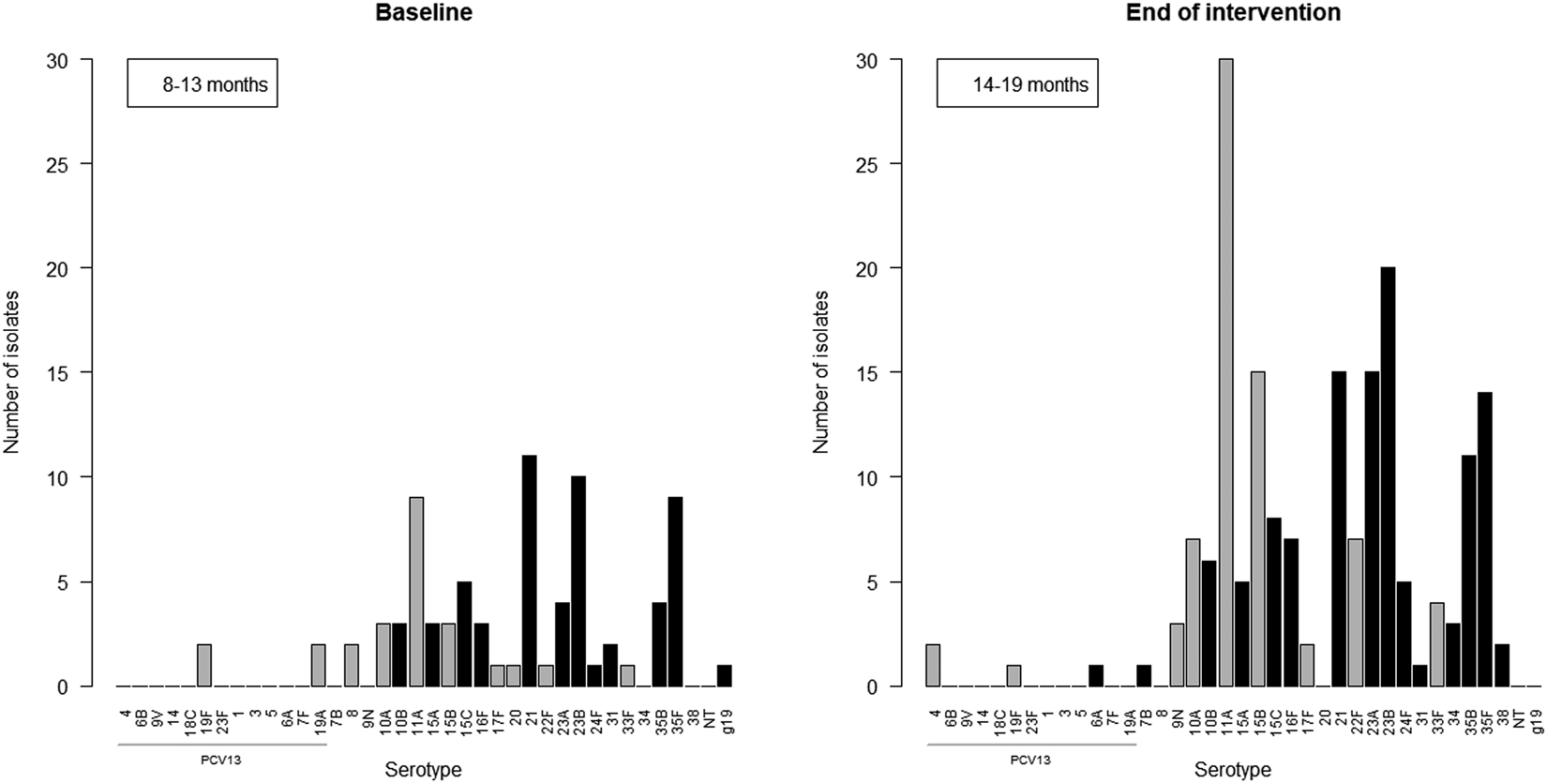
Carriage study and serotype distribution in Danish children at baseline and at the end of intervention. Grey color indicates PPV23-serotypes, black is non-PPV23 serotypes. Serotypes are listed with PCV7-serotypes first, then PCV13-serotypes followed by non-PCV13-serotypes.

The 81 isolates found at baseline belonged to 21 different serotypes and 1 serogroup (where serotyping was not possible) (Fig. 1). The most common serotype isolated at baseline was serotype 21 constituting 13.6% of all isolates. Serotype 23B, 35F and 11A were also commonly isolated, constituting 12.3%, 11.1% and 11.1% of all isolates respectively (Fig. 1). The only PCV13-serotypes isolated at baseline were 19A and 19F, which both constituted 2.5% of all isolates.

At the end of intervention we found 185 isolates belonging to 24 different serotypes. Serotype 11A was the most common serotype constituting 16.2% of all isolates. Serotype 23B, 15B, 21, 23A and 35F were also common, constituting 10.8%, 8.1%, 8.1%, 8.1% and 7.6% of all isolates respectively (Fig. 1). Serotype 4, 19F and 6A were the only PCV13-serotypes found at the end of intervention, only constituting 1.1%, 0.5% and 0.5% of all isolates respectively (Fig. 1).

Serotype 8 was only found in two children at baseline (Fig. 1).

### Risk factors

Sex did not influence the carriage rate of *S. pneumoniae* significantly (p = 0.280 at baseline and p = 0.187 at end of intervention) (Tables 3 and 4).

**Table 3:** Potential risk factors for pneumococcal carriage in the baseline group and at the end of study. The table shows number of carriers, total number of children, carriage rates, p-values, odds ratios (OR) and confidence intervals (CI).

		Number of carriers (total number of children)	Carriage rate % (95% CI)	OR (95% CI)*	p-value*
**Baseline**					
Sex	Females	32 (140)	22.9 (15.9–29.8%)	1.00	1.00
	Males	42 (145)	29.0 (21.6–36.3%)	1.375 (0.781–2.436)	0.280
Siblings <5 years	No**	14 (148)	9.5 (4.7–14.2%)	1.00	1.00
	Yes	49 (94)	52.1 (42.0–62.2%)	10.299 (5.043–22.217)	<0.001
Breastfeeding at baseline examination	No	32 (150)	21.3 (14.8–27.9%)	1.00	1.00
	Yes	42 (135)	31.1 (23.3–38.9%)	1.662 (0.944–2.950)	0.078
Respiratory infections during intervening period ***	No	N/A	N/A	N/A	N/A
	Yes	N/A	N/A	N/A	N/A
Bronchitis	No	N/A	N/A	N/A	N/A
	Yes	N/A	N/A	N/A	N/A
Pneumonia	No	N/A	N/A	N/A	N/A
	Yes	N/A	N/A	N/A	N/A
Otitis media	No	N/A	N/A	N/A	N/A
	Yes	N/A	N/A	N/A	N/A
Systemic antibiotic during intervening period	No	N/A	N/A	N/A	N/A
	Yes	N/A	N/A	N/A	N/A
Passive smoking	No	67 (247)	27.1 (21.6–32.7%)	1.00	1.00
	Yes	7 (38)	18.4 (6.1–30.7%)	0.608 (0.215–1.495)	0.322
Living with a dog or cat	No	56 (221)	25.3 (19.6–31.1%)	1.00	1.00
	Yes	14 (47)	29.8 (16.7–42.9%)	1.249 (0.574–2.608)	0.584

^*^Odds ratios, confidence intervals and p-values were calculated by two-tailed Fisher’s Exact Test. ^**^No siblings at all ^***^including bronchitis, pneumonia, sore throat, sinusitis, croup and otitis media N/A = not available.

CI: confidence interval, OR: odds ratio.

**Table 4:** Potential risk factors for pneumococcal carriage in the baseline group and at the end of intervention. The table shows number of carriers, total number of children, carriage rates, p-values, odds ratios (OR) and confidence intervals (CI).

		Number of carriers (total number of children)	Carriage rate % (95% CI)	OR (95% CI)*	p-value*
**End of intervention**					
Sex	Females	90 (126)	71.4 (63.5–79.3%)	1.00	1.00
	Males	84 (132)	63.6 (55.4–71.8%)	0.701 (0.400–1.222)	0.187
Siblings <5 years	No**	89 (130)	68.5 (60.5–76.4%)	1.00	1.00
	Yes	59 (88)	67.0 (57.2–76.9%)	0.938 (0.506–1.748)	0.883
Breastfeeding at baseline examination	No	89 (134)	66.4 (58.4–74.4%)	1.00	1.00
	Yes	85 (124)	68.5 (60.4–76.7%)	1.102 (0.633–1.922)	0.791
Respiratory infections during intervening period***	No	97 (139)	69.8 (62.2–77.4%)	1.00	1.00
	Yes	77 (119)	64.7 (56.1–73.3%)	0.795 (0.456–1.384)	0.425
Bronchitis	No	164 (236)	69.5 (63.6–75.4%)	1.00	1.00
	Yes	10 (22)	45.5 (24.6–66.3%)	0.367 (0.135–0.975)	0.031
Pneumonia	No	149 (222)	67.1 (60.9–73.3%)	1.00	1.00
	Yes	25 (36)	69.4 (54.4–84.5%)	1.113 (0.495–2.650)	0.85
Otitis media	No	127 (182)	69.8 (63.1–76.5%)	1.00	1.00
	Yes	47 (76)	61.8 (50.9–72.8%)	0.703 (0.387–1.285)	0.244
Systemic antibiotic during intervening period	No	113 (163)	69.3 (62.2–76.4%)	1.00	1.00
	Yes	61 (95)	64.2 (54.8–73.9%)	0.795 (0.45–1.41)	0.411
Passive smoking	No	152 (226)	67.3 (61.1–73.4%)	1.00	1.00
	Yes	22 (32)	68.8 (52.7–84.8%)	1.071 (0.458–2.669)	1
Living with a dog or cat	No	141 (200)	70.5 (64.2–76.8%)	1.00	1.00
	Yes	24 (42)	57.1 (42.2–72.1%)	0.559 (0.268–1.181)	0.103

^*^Odds ratios, confidence intervals and p-values were calculated by two-tailed Fisher’s Exact Test. ^**^No siblings at all ^***^including bronchitis, pneumonia, sore throat, sinusitis, croup and otitis media N/A = not available.

CI: confidence interval, OR: odds ratio.

Having siblings <5 years significantly increased the carriage rate of *S. pneumoniae* in children at baseline (p < 0.001), but did not influence the carriage rate of children at the end of intervention (p = 0.883) (Tables 3 and 4).

Breastfeeding at the time of baseline examination, receiving systemic antibiotics during the intervention period, exposure to passive smoking or living with a dog or cat did not influence the carriage rate of *S. pneumoniae* (Tables 3 and 4).

**Figure 2:**
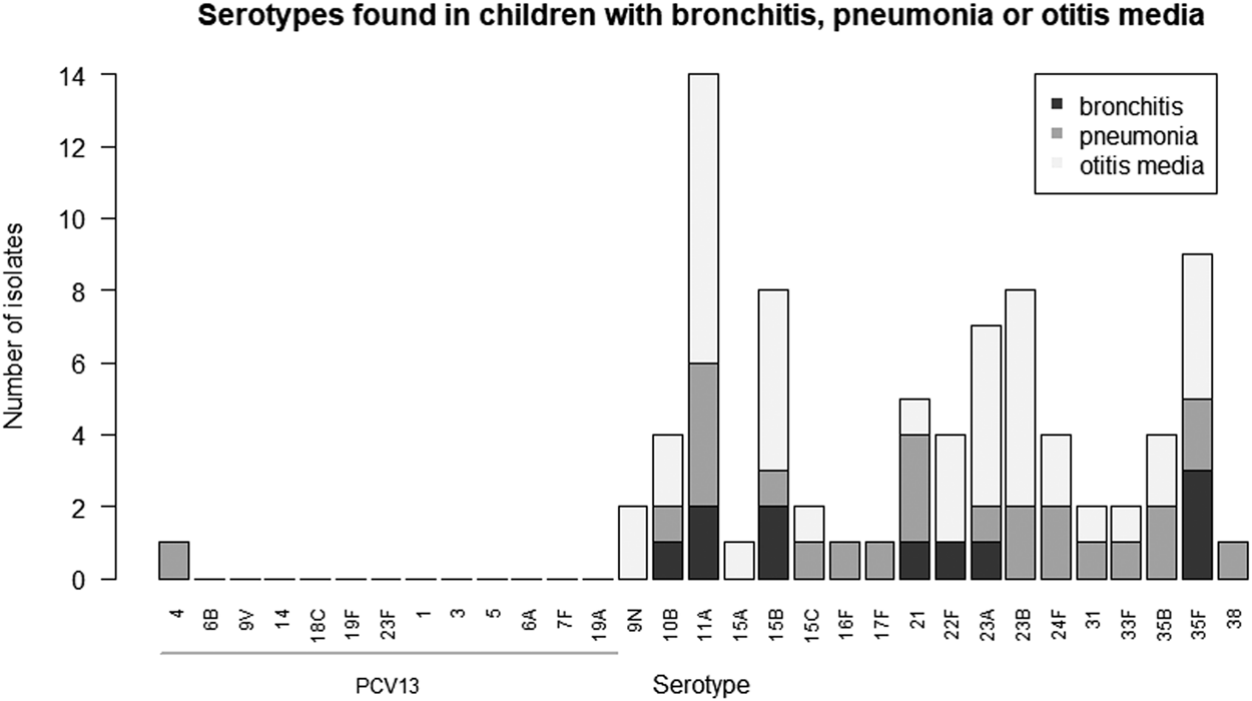
Serotypes and number of isolates found in children who have experienced bronchitis, pneumonia and/or otitis media in the intervening period between baseline and the end of intervention. Infections were at any time during the intervening period. Serotypes are mentioned as PCV7-serotypes first, then PCV13-serotypes followed by non-PCV13-serotypes.

We examined if children with respiratory infections during the intervening period had an increased risk of pneumococcal carriage, but no significant difference in carriage rate was observed between children having had respiratory infections or children not affected by respiratory infections (p = 0.425) (Table 4). However, children who had experienced bronchitis during intervention seemed to have a lower carriage rate compared to children who had not experienced bronchitis (p = 0.031), but this result may not be reliable due to the low number of children in the cohort (Table 4). Serotype distribution in children who have experienced bronchitis, pneumonia or otitis media is shown in Fig. 2.

### Registered invasive pneumococcal diseases in Denmark

**Figure 3:**
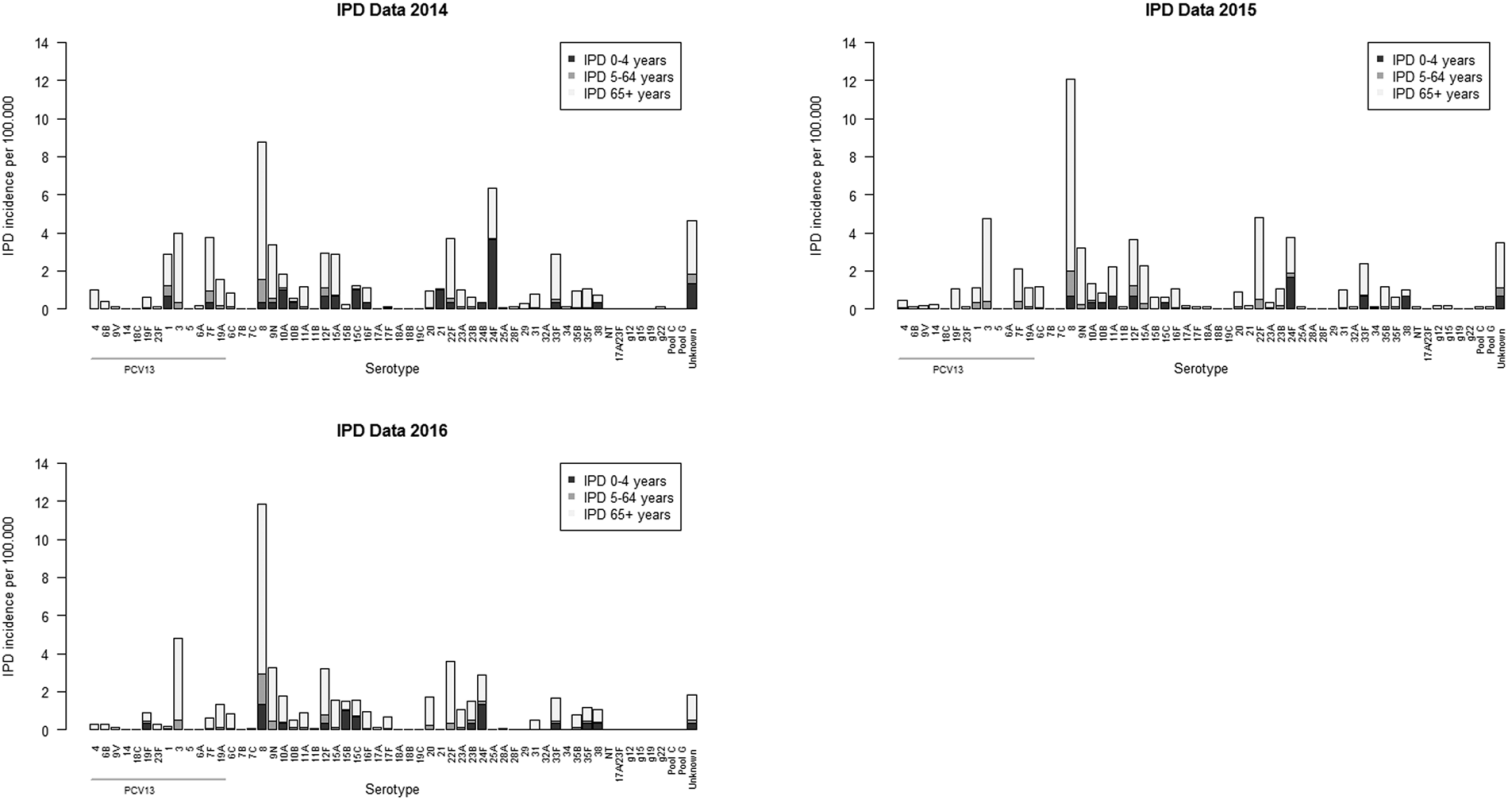
Serotypes found in patients with IPD aged 0–4 years, 5–64 years and 65+ years. The figure shows IPD incidences per 100.000. Serotypes are mentioned as PCV7-serotypes first, then PCV13-serotypes followed by non-PCV13-serotypes. Data were obtained from the Danish laboratory surveillance system at the National Neisseria and Streptococcus Reference Laboratory (NSR), Statens Serum Institut (SSI) as described by Slotved *et al*. (2016)^14,15^.

Data regarding IPD-causing serotypes in Denmark were obtained from the Danish laboratory surveillance system as mentioned in the methods section and Fig. 3 shows the data. Serotype 24F caused most of the IPDs in Denmark among 0–4 years old children in 2014 and 2015. Other commonly isolated serotypes from this patient group was 10A, 15C and 21 in 2014 and 8, 12F, 33F, 11A and 38 in 2015. In 2016 serotype 8 was causing most of the IPDs among 0–4 years old and the second most common serotype isolated was 24F.

Among 5–64 years old serotype 8 was causing most of the registered IPDs in both 2014–2016. Among 65+ years old serotype 8 was also causing most of the IPD cases in Denmark in 2014–2016. Serotype 3, 7F, 9N, 22F and 12F were also common serotypes isolated in patients aged 65+ years in 2014–2016.

## Discussion

To our knowledge this is the first carriage study of *S. pneumoniae* in children after the introduction of PCV in Denmark in 2007.

Oral administration of probiotics to children aged 8–13 months for 6 months had no effect on the carriage of pneumococcal serotypes as tested in two different years (2014 and 2015). Furthermore, we found an overall carriage rate in children attending daycare (end of intervention) of 67.4% as compared to a carriage rate of 69% before the introduction of PCV7 into the Danish Childhood Immunization Program^9,19^. Thus, PCV-use seems not to have influenced the overall carriage rate in children.

Pneumococcal vaccine-serotypes such as 6A, 23F, 14 and 19F, were frequently observed as carried serotypes in Denmark prior to the introduction of PCVs^9^. Our study revealed that vaccine-serotypes have almost disappeared in Danish children aged <2 years after the introduction of PCV13 and have been replaced by non-vaccine serotypes such as 11A, 23B, 23A, 21, 15B, 15C, 35F and 35B (Fig. 1) as observed in other countries^7,10,20–22^.

Living with siblings <5 years of age significantly increased the risk of pneumococcal carriage before starting in daycare (baseline) (p < 0.001) (Table 3). This has also been observed by others^10–12^. We found no significant difference in carriage rate of *S. pneumoniae* among children receiving probiotics and children receiving placebo (Table 1). Nasopharyngeal carriage of *S. pneumoniae* is considered a prerequisite for developing pneumococcal associated diseases such as otitis media, pneumonia, bronchitis and other respiratory infections^5,23,24^. Wong *et al*. reported that the probiotic LGG inhibits the adherence of *S. pneumoniae* to human epithelial cells *in vitro*^25^. Clinical studies investigating the effect of administrating probiotics on the incidence of respiratory tract infections have shown inconclusive results. Laursen *et al*.^17^ did not find LGG and BB-12 to reduce the number of days absent from daycare because of respiratory or gastrointestinal infections in the same children as included in our study, and the probiotics did not affect the endogenous gut microbiota composition in those children^26^. A Swedish study involving 248 healthy children aged 1–5 years and attending daycare showed a significantly lower incidence of otitis media in children who ingested milk with 10^7^ CFU/mL *Lactobacillus rhamnosus* LB21 compared to placebo^27^. However, a study including 309 otitis media-prone children aged 10 months to 6 years showed no significant difference in the incidence of otitis media or nasopharyngeal carriage of *S. pneumoniae* between the group receiving capsules containing probiotics (8–9 × 10^9^ CFU/capsule) and the placebo group^28^. Others found that LGG and *Bifidobacterium lactis* ingested orally reduced the risk of otitis media and recurrent respiratory infections in children <2 years^29^, as did milk containing LGG in healthy children aged 1–6 years^30^, while Skovbjerg *et al*.^31^ report that nasal delivery of the probiotic *Lactobacillus rhamnosus* did not affect nasopharyngeal pneumococcal carriage in children aged 1–8 years with secretory otitis media^31^.

The carriage rate at the end of intervention was significantly higher than at baseline (p < 0.001) (Table 1). The age difference between these two groups was small (8–13 months at baseline and 14–19 months at the end of intervention), and the use of probiotics during intervention did not affect the carriage rate (Table 1), hence these factors could not be the reason for this difference. Baseline samples were collected during autumn season, while samples at the end of intervention were collected 6 months later (spring season), however the effect of seasonality on the carriage of *S. pneumoniae* is not clear^4^. The children were not attending daycare at baseline, while all the children attended daycare at the end of intervention. Attending daycare is considered an important risk factor in relation to acquisition of pneumococcal carriage^9,11,12^, hence the higher carriage rate at the end of intervention might be explained by daycare attendance.

Since we consider our data non-parametric and the general choice of statistical test within this field is two-tailed Fishers exact test we have used the univariate analysis by two-tailed Fisher’s Exact Test to calculate the risk factors. This is not an age-matched group however the range of the age group is very small and we have not found studies showing such small age range to chance the carriage rate but external factors do chance the carriage rate such as daycare attendance^9,11,12^. Most studies include children aged 0–24 months as one group^3,4,7,9,20,29^ and since the children included in our study are aged 8–19 months we believe the age range is not significant. The main differences between the baseline group (aged 8–13 months) and end of intervention group (aged 14–19 months) is daycare attendance and whether the children have received probiotics or not.

It is believed that children with high colonization rates of *S. pneumoniae* are the key transmitters of non-vaccine serotypes causing IPD to other age groups^6,8,22,32^. IPD caused by vaccine-serotypes has declined significantly after the implementation of PCV in children, while IPD caused by non-vaccine serotypes has increased^8,14,33^. This is consistent with the decline in carriage of vaccine-serotypes and an increase in carriage of non-vaccine serotypes in children which is supported here (Fig. 1) and by others^7,20,22,34^. Despite the use of effective vaccines, serotypes 3, 7F and 19A, which are included in PCV13, are still causing IPD among elderly in Denmark^13,14^ (Fig. 3). The low carriage rate of serotype 8 among 8–19 months old Danish children in our study contrasts the high prevalence of serotype 8 seen among elderly patients with IPD in Denmark. The other 5 most common serotypes (3, 22F, 9N, 12F and 7F) isolated from elderly with IPD in 2014–2016 were either not found or found with low prevalence (Figs 1 and 3). It has been assumed that immunization of infants alone would result in herd immunity and hence protect adults and elderly against disease caused by vaccine-serotypes^21,35,36^. Our findings suggest that children aged 8–19 months are not the main reservoir of serotypes causing IPD in elderly, but suggest that other age groups need to be considered as main carriers of some pneumococcal serotypes.

We detected that children aged 8–19 months were carriers of serotypes 24F, 10A, 11A, 15A, 15B, 21, which cause IPD in children aged 0–4 years (Figs 1 and 3).

The limitation of this study is that it included a narrow age group of children (8–19 months) and that the study only included children living in the capital region of Denmark. The strength of the study is, that we have examined very young children who usually are difficult to collect swab samples from and were able to obtain detailed background information regarding the participants.

In conclusion, this study revealed that PCV-serotypes have disappeared in children and been replaced by non-PCV serotypes after the introduction of PCVs in Denmark. The probiotics LGG and BB-12 did not prevent carriage of *S. pneumoniae* in children attending daycare. Having a sibling <5 years before daycare increased the risk of pneumococcal carriage significantly. Children aged 8–19 months are carriers of pneumococcal serotypes causing IPD in children aged 0–4 years. However, the children seem not to be carriers of serotypes causing the majority of IPD cases in elderly in Denmark.

## Electronic supplementary material

Supplementary Information
