# Supplementary material for: Probiotics and carriage of Streptococcus pneumoniae serotypes in Danish children, a double-blind randomized controlled trial
Source: Sci Rep. 2018 Oct 15;8:15258. doi: 10.1038/s41598-018-33583-9 (PMC6189121; doi:10.1038/s41598-018-33583-9)
Supplement: Supplementary file 1 — Supplementary Information [file 41598_2018_33583_MOESM1_ESM.pdf]

We updated the design of this site on December 18, 2017. Learn more.

NIH U.S. National Library of Medicine
ClinicalTrials.gov

Find Studies
About Studies
Submit Studies
Resources
About Site

Effect of Probiotics in Reducing Infections and Allergies in Young Children Starting Daycare (ProbiComp)

The safety and scientific validity of this study is the responsibility of the study sponsor and investigators.
Listing a study does not mean it has been evaluated by the U.S. Federal Government. Read our disclaimer for details.

ClinicalTrials.gov Identifier:
NCT02180581

Recruitment Status : Completed
First Posted : July 2, 2014
Last Update Posted : August 24, 2016

Sponsor:
University of Copenhagen

Collaborators:
The Danish Council for Strategic Research
Chr Hansen
Technical University of Denmark
University of Bergen
Odense University Hospital
Statens Serum Institut

Information provided by (Responsible Party):
Arne Astrup, University of Copenhagen

Study Details | Tabular View | No Results Posted | Disclaimer
How to Read a Study Record

Study Description

Go to

Brief Summary:

The aim of the intervention is to examine the effect of a combination of the two bacterial strains BB-12 and LGG, provided for 6 month, on the prevalence of infections and allergic manifestations in small children, and how a combination of BB-12 and LGG affects the immune system, gastrointestinal tract and the microbiota. Children are enrolled during 2 winter seasons.

Condition or disease	Intervention/treatment
Days Absent From Daycare	Dietary Supplement: Probiotic (2 * 10^9 cfu/day)
Respiratory Tract Infections	Dietary Supplement: Placebo
Gastrointestinal Infections	
Allergy	

Study Design

Go to

Study Type :	Interventional (Clinical Trial)
Actual Enrollment :	290 participants
Allocation:	Randomized
Intervention Model:	Parallel Assignment
Masking:	Quadruple (Participant, Care Provider, Investigator, Outcomes Assessor)
Primary Purpose:	Prevention
Official Title:	Effect of Probiotics in Reducing Infections and Allergies in Young Children Starting Daycare
Study Start Date :	August 2014
Primary Completion Date :	June 2016
Study Completion Date :	June 2016

Resource links provided by the National Library of Medicine

NIH NLM

MedlinePlus related topics: Allergy

U.S. FDA Resources

Arms and Interventions

Go to

Arm	Intervention/treatment
Experimental: Probiotic (2 * 10^9 cfu/d)<br>Daily intake of bifidobacterium animalis ssp. Lactis (BB12) and Lactobacillus Rhamnosus GG (LGG) in a dosage of 10^9 cfu/day of each strain. The probiotics are provided as powder in a sachet, and can be added to food or drink	Dietary Supplement: Probiotic (2 * 10^9 cfu/day)<br>Combination of two probiotics (2 * 10^9 cfu/day) or probiotics for 6 months<br>Other Names:<br>• bifidobacterium animalis ssp. Lactis (BB12), 10^9 cfu/day<br>• Lactobacillus Rhamnosus GG (LGG), 10^9 cfu/day
Placebo Comparator: Placebo<br>provided as powder in a sachet, and can be added to food or drink	Dietary Supplement: Placebo<br>Placebo for 6 months

Outcome Measures

Go to

Primary Outcome Measures :

1. Number of days absent from daycare due to respiratory and gastrointestinal infections [Time Frame: up to 6 month]

   Recorded weekly by the parents using web-based questionnaires

Secondary Outcome Measures :

1. Acute upper respiratory tract infections [Time Frame: Up to 6 month]

   Number of days with acute upper respiratory tract infections (URTI); Number of children with at least 1 episode of URTI; Number of children with at least 3 episodes of URTI; Number of URTI episodes/child/year; Duration of URTI episodes (days);

   Additionally,symptoms of cold (defined as 2 days with runny/stuffy nose or cough)

   Recorded by the parents daily/weekly using web-based questionnaires.

2. Acute lower respiratory infections [Time Frame: Up to 6 month]

Number of children with at least 1 episode of a lower respiratory tract infection (LRTI).

LRTI include bronchitis and pneumonia

Recorded by the parents weekly using web-based questionnaires.

3. Gastrointestinal infections [Time Frame: Up to 6 month]

   Number of children with at least 1 episode of diarrhea; Duration of episodes with diarrhea(days); Number of episodes/child/year with diarrhea; Number of days with vomiting.

   Recorded by the parents daily using web-based questionnaires

4. Fever [Time Frame: Up to 6 month]

   Number of days with fever

   Recorded by the parents using web-based questionnaires

5. Antibiotic use [Time Frame: Up to 6 month]

   Number of treatments with antibiotics during the intervention period

   Recorded weekly by the parents using web-based questionnaires

6. Allergies [Time Frame: Up to 6 month]

   Number of children developing allergies during the intervention period (asthma, allergic rhinitis, atopic dermatitis, food allergies); Age for diagnosis of allergies; Use of medication due to allergies.

   Recorded by the parents using web-based questionnaires

7. Absence from day care due to illness other than infections [Time Frame: Up to 6 month]

   Number of days the child is absent from day care due to illness, which is not due to infections.

   Recorded by the parents weekly using web-based questionnaires

8. Parental absence from work due to illness of the child [Time Frame: Up to 6 month]

   Number of days a parent is absent from work due to illness of the child (infections and other illnesses, respectively)

   Recorded by the parents weekly using web-based questionnaires

9. Medical visits [Time Frame: Up to 6 month]

   Number of visits to a doctor due to infections and other illnesses, respectively.

   Recorded by the parents using web-based questionnaires

10. Change from baseline in Thymus size [Time Frame: At baseline and after 6 mo (end of intervention)]

    Change in thymic size during intervention period will be evaluated using ultrasound

Other Outcome Measures:

1. Change from baseline in biological markers for the immune system [Time Frame: At baseline and after 6 mo]

   Analysis of biological material investigating the effect on the immune system by biological markers .

2. Change from baseline in biological markers for gastro-intestinal tract [Time Frame: At baseline and after 6 mo]

   Analysis of biological material investigating the effect on the gastrointestinal tract.

3. Change from baseline in Biological markers of allergy [Time Frame: At baseline and after 6 mo]

   analysis of biological samples investigating the effect on allergy by biological markers at baseline and at 6 mo

Eligibility Criteria

Go to

Information from the National Library of Medicine

NIH NLM

Choosing to participate in a study is an important personal decision. Talk with your doctor and family members or friends about deciding to join a study. To learn more about this study, you or your doctor may contact the study research

staff using the contacts provided below. For general information, Learn About Clinical Studies.

Ages Eligible for Study:	8 Months to 14 Months (Child)
Sexes Eligible for Study:	All
Accepts Healthy Volunteers:	Yes

Criteria

Inclusion Criteria:

- Expected to start daycare between the age of 8-14 month
- Intervention start 0 days to 12 weeks before starting daycare
- Expected to start daycare from september to february (both month included)
- Single born

Exclusion Criteria:

- Exclusion Criteria:
- Children born before 37th gestational week
- Children with a birth weight < 2500 g
- Children suffering from severe chronic illness
- Children receiving regular medication
- Children who have received antibiotics within a month before intervention start
- Children whose parents do not speak Danish

Contacts and Locations

Go to

Information from the National Library of Medicine

NIH NLM

To learn more about this study, you or your doctor may contact the study research staff using the contact information provided by the sponsor.

*Please refer to this study by its ClinicalTrials.gov identifier (NCT number): **NCT02180581***

Locations

Denmark

Section of Paediatric and International Nutrition, Department of Nutrition, Exercise and Sports, Fa
Frederiksberg, Denmark, 1958

Sponsors and Collaborators

University of Copenhagen

The Danish Council for Strategic Research

Chr Hansen

Technical University of Denmark

University of Bergen

Odense University Hospital

Statens Serum Institut

More Information

Go to

Responsible Party:	Arne Astrup, Professor, University of Copenhagen
ClinicalTrials.gov Identifier:	NCT02180581 History of Changes
Other Study ID Numbers:	H-4-2014-032<br>H-4-2014-032 (Other Grant/Funding Number: The Danish Council for Strategic Research)
First Posted:	July 2, 2014 Key Record Dates
Last Update Posted:	August 24, 2016
Last Verified:	August 2016

Keywords provided by Arne Astrup, University of Copenhagen:
Infections
Allergy
Immune function
Microbiota
Infant

Additional relevant MeSH terms:
Infection
Communicable Diseases
Hypersensitivity
Respiratory Tract Infections
Immune System Diseases
Respiratory Tract Diseases
